# Diesel Engine Exhaust Initiates a Sequence of Pulmonary and Cardiovascular Effects in Rats

**DOI:** 10.1155/2010/206057

**Published:** 2010-10-31

**Authors:** Ingeborg M. Kooter, Miriam E. Gerlofs-Nijland, A. John F. Boere, Daan L. A. C. Leseman, Paul H. B. Fokkens, Henri M. H. Spronk, Kim Frederix, Hugo ten Cate, Ad M. Knaapen, Hendrik J. Vreman, Flemming R. Cassee

**Affiliations:** ^1^Department of Environment, Health and Safety, TNO Built, Environment and Geosciences, Princetonlaan 6, 3584 CB Utrecht, The Netherlands; ^2^Centre for Environmental Health Research, National Institute for Public Health and the Environment, 3720 BA Bilthoven, The Netherlands; ^3^Department of Internal Medicine, Laboratory of Clinical Thrombosis and Haemostasis, Cardiovascular Research Institute Maastricht, Maastricht University Medical Center, 6229 ER Maastricht, The Netherlands; ^4^Department of Health Risk Analysis and Toxicology, Nutrition and Toxicology Research Institute (NUTRIM), Maastricht University, 6229 ER Maastricht, The Netherlands; ^5^Organon, Schering Plough, 5342 CC Oss, The Netherlands; ^6^Division of Neonatal & Developmental Medicine, Department of Pediatrics, Stanford University Medical Center, Stanford, CA 94305-5208, USA

## Abstract

This study was designed to determine the sequence of events leading to cardiopulmonary effects following acute inhalation of diesel engine exhaust in rats. Rats were exposed for 2 h to diesel engine exhaust (1.9 mg/m^3^), and biological parameters related to antioxidant defense, inflammation, and procoagulation were examined after 4, 18, 24, 48, and 72 h. This in vivo inhalation study showed a pulmonary anti-oxidant response (an increased activity of the anti-oxidant enzymes glutathione peroxidase and superoxide dismutase and an increase in heme oxygenase-1 protein, heme oxygenase activity, and uric acid) which precedes the inflammatory response (an increase in IL-6 and TNF-*α*). In addition, increased plasma thrombogenicity and immediate anti-oxidant defense gene expression in aorta tissue shortly after the exposure might suggest direct translocation of diesel engine exhaust components to the vasculature but mediation by other pathways cannot be ruled out. This study therefore shows that different stages in oxidative stress are not only affected by dose increments but are also time dependent.

## 1. Introduction

Epidemiological studies have shown associations between daily changes in air pollution such as particulate matter (PM) and cardiopulmonary morbidity and mortality [1, 2]. Although the relative risk estimates are small, there is a serious public health concern because of the large number of people exposed and the existence of high risk groups, such as the elderly and people with cardiopulmonary diseases.

PM can be considered a complex chemical mixture that may have various effects on pulmonary and cardiovascular tissues depending on its physicochemical properties. There is, however, a need to obtain better insight into the plausibility of the PM-associated health effects for risk assessment purposes. This information can be obtained from studies focusing on the processes underlying the health effects of PM. Several mechanisms have been postulated and include injury of pulmonary epithelial tissue, inflammation, and oxidative stress response. In addition, extrapulmonary tissues, such as those of the cardiovascular system, may be injured either directly via translocation of particles or soluble components into the circulation or indirectly via pulmonary inflammatory effects. Such vascular effects may translate into a prothrombotic phenotype [3]. Among the suggested mechanisms, oxidative stress may play a key role in causing the adverse cardiopulmonary health effects of ambient PM [4, 5]. An important function of cellular homeostasis is to maintain the balance between reactive oxygen species (ROS) and anti-oxidant defense. Oxidative stress can be caused by ROS production overwhelming the anti-oxidant protection, leading to tissue damage [6]. Oxidative stress can be induced by endogenous, as well as exogenous factors. Exogenous sources of ROS include environmental factors such as smoking, diet, and exposure to air pollution such as PM. PM can induce oxidative stress in several ways. For example, there are several soluble transition metals on the surface of particles, such as iron and copper, that can generate hydroxyl radicals through the Fenton reaction [7, 8]. Furthermore, alveolar macrophages are known to ingest and remove inhaled particles from the lungs, and neutrophils also respond to these particles [9]. Activation of macrophages results in a release of cytokines, as well as ROS through a so-called respiratory burst [10]. In addition, the reactive organic soluble substances of particles, which can consist of several redox-active quinones, may play a role due to their electrophilic properties [11]. These can also be generated during metabolism of several polycyclic aromatic hydrocarbons (PAHs) by cytochrome P-450 (CYP1A1) [12]. Many have studied the process of oxidative stress caused by particles *in vitro* as well as *in vivo*, both in rodents as well as in humans [13–15]. However, there is a clear lack in information on the sequence of events that occur in time both in the pulmonary and cardiovascular tissues upon inhalation to a controlled atmosphere containing particles with expected high oxidative potential [16]. The present study was designed to determine the sequence of events leading to cardiopulmonary effects following an acute inhalation of diesel engine exhaust (DEE) in rats.

## 2. Methods

### 2.1. Animal Housing and Exposure

Male Fischer F344 rats (9 weeks old) were obtained from Charles River (Germany). Animals were weighted and randomly allocated. The animals were housed in macrolon cages (type III; two rats per cage) containing toy enrichment on coarse foundation (Abedd LTE E-001). The animals were fed with RMH-GS (Hope-Farms) pellets and tap water via bottles. Animals were trained in nose-only tubes for 5 days, one hour per day, in the week prior to exposure.

Animals were placed individually in nose-only tubes and exposed for two hours to fresh diluted DEE, with mass concentration of 1.9 mg/m^3^. DEE was generated by mixing a small flow out of DEE from the idling (1500 rpm) engine (type F3M2011 Deutz Ag, Köln, Germany) of a 35 KVA generator (Bredenoord, Apeldoorn, The Netherlands) into filtered ambient air in a mixing/buffering chamber upstream of the two parallel nose-only units. The EN 950 diesel used (Shell Gasoline ultra low sulpher, 1697NL02) contained maximum 50 mg/kg sulphur. Control animals were exposed to filtered ambient air in a similar system. Flows through both parallel nose-only units were approximately 40 liters per minute (Lpm). Total flow for measurements was approximately 10 Lpm. Total flow entering the mixing/buffering chamber was approximately 120 Lpm. Experiments were approved by the Ethical Review Committee of the National Institute for Public Health and the Environment, Bilthoven, The Netherlands.

### 2.2. Diesel Engine Exhaust Exposure Characteristics

The size distribution of the particles in the diluted DEE was determined once prior to the exposure period with a Multi-Orifice Impactor (MOI 100, MSP corp., Minneapolis MN, USA) using the following stages: <0.18, 0.18 < 0.32, 0.32 < 0.56, 0.56 < 1.0, 1.0 < 1.8, 1.8 < 2.5, ≥ 2.5 *μ*m. The mass concentration was measured with a probe inside the mixing/buffering chamber through collection on two parallel 47-mm filters (Polytetrafluorethylene, PTFE) type R2PL047 (Pall, USA). A charcoal sampler tube was placed downstream of one of the Teflon filters to collect volatile organic compounds (VOCs) at a flow rate of 5 Lpm. DEE particles were sampled with a probe inside the mixing/buffering chamber on a quartz filter for time integrated mass concentrations as well as for EC/OC analysis. Temperature and relative humidity were measured during exposures with Humbug data loggers inside the nose-only units (exposure/control) and inside the animal housing room. Temperature and relative humidity were 21 ± 2°C and 50% ± 20%, respectively. NOx (Nitrogen Oxides analyzer model 42W, Thermo Electron corp., Hopkinton MA, USA), CO (Carbon Monoxide analyzer model 9830B CO, Lear Siegler Measurement Controls corp., Englewood CO, USA) and the particle number (CPC 3022, TSI inc., St Paul MN, USA) and mass concentration (DataRam 2000, MIE inc., Billerca MA, USA) were continuously measured with a probe inside the mixing/buffering chamber and recorded either on a paper chart or stored internal (DataRam 2000). The composition and the concentrations of the diluted DEE are given in Table 1. The levels of sulfates and nitrates in the DEE exposure were relatively low and not expected to contribute significantly to the concentrated particle atmospheres. Particle numbers were dominated by ultrafine particles (0.1 *μ*m). Metal and PAH composition, determined via Q-ICP-MS and HPLC-fluorescence, respectively, of the diluted DEE is indicated in Table 2.

### 2.3. Necropsy

At 4, 18, 24, 48, and 72 h postexposure animals were weighed, and anaesthetized with a mixture of ketamine (100 mg/mL, Aesculaap, Boxtel, The Netherlands) and xylazine (20 mg/mL, Bayer, Leverkusen, Germany) at a ratio of 10 : 8. The anesthetic was injected i.p. (1.5 mL/kg body weight), resulting in a dose of 83 mg ketamine/kg body weight and 13 mg xylazine/kg body weight. Animals were sacrificed by exsanguinations via the abdominal aorta and at least 5 mL blood was collected. Saline perfusion of the lungs was performed via the right cardiac ventricle to remove blood. The heart and thoracic aorta were excised, weighed, and snap-frozen in liquid nitrogen. The left bronchus was pinched off, the left lung was dissected, weighed and immediately frozen in liquid nitrogen. Then the right lung was lavaged (three in/out lavages using same fluid) with a volume of saline corresponding with 27 mL/kg body weight at 37°C to obtain bronchoalveolar lavage fluid (BALF). The recovered BALF was placed on ice until further processing. The right lung was dissected, weighed and immediately frozen in liquid nitrogen.

### 2.4. Bronchoalveolar Lavage Fluid Analysis

The BALF from each animal was centrifuged at 400× g for 10 min at 4°C. The cell-free fluid from the lavage was used for measurements of cellular toxicity, inflammation, and oxidative stress. The pellet from the lavage was resuspended in 1 mL saline and used for preparation of cytospins for differential cell counts.

#### 2.4.1. Cell Differentiation

Cytospin slides were made in duplicate for differential cell counts and stained according to May-Grünwald and Giemsa. Per cytospin slide, 200 cells were counted (total of 400 cells per exposure) and the percentage of each cell type (macrophages, PMN, eosinophilic granulocytes and lymphocytes) was determined.

#### 2.4.2. Biochemical Characterization

Lactate dehydrogenase (LDH), alkaline phosphatase (ALP), N-acetyl glucosamidase (NAG), and uric acid (UA) were determined using a commercially obtained reagent kit (Roche Nederland BV, Almere, The Netherlands). LDH was measured as a marker for cytotoxicity ALP and was measured as a marker for epithelial type II cell damage. NAG was measured as a marker for macrophage activation, albumin as a marker for epithelial damage of the alveoli and in addition, the anti-oxidant UA was measured as a marker for oxidative stress. HO-1 as a marker for oxidative stress was measured using a commercially available ELISA kit (StressGen, ITK Diagnostics BV, Uithoorn, The Netherlands).

#### 2.4.3. Cytokines

The inflammatory mediators, TNF-*α* and interleukin-6 (IL-6) were determined using commercially available enzyme-linked immunosorbent assay (ELISA) kit (Biosource, Etten-Leur, The Netherlands). Clara cell protein (CC16) was determined by ELISA using anti-CC16 antiserum and polyclonal anti-CC16 antibody kindly provided by Prof. G. Singh (VA Medical Center and University of Pittsburgh School of Medicine, Pittsburgh, USA). The CC16 standard was kindly provided by Prof. A. Bernard (Catholic University of Louvain, Brussels, Belgium).

### 2.5. Lung Homogenate Analysis

Frozen apical lob of the right lung was homogenized in 120 mM KCl, 30 mM phosphate buffer (pH 7.2), containing protein inhibitors (1 *μ*g/mL leupeptin, 1 *μ*g/mL aprotinin, 10 *μ*g/mL soybean trypsin inhibitor, 1 *μ*g/mL pepstatin, and 0.5 mM phenylmethylsulfonyl fluoride) at 4°C. The suspensions were centrifuged at 600× g for 10 min at 4°C to remove nuclei and cell debris. The pellets were discarded and the supernatants were used as homogenates according to Rhoden et al. [17].

#### 2.5.1. Oxidative Stress Parameters

Malondialdehyde (MDA) was measured in the lung homogenate supernatant using HPLC with fluorescence detection (Varian Associates, Middelburg, The Netherlands). GPx and SOD were measured on an autoanalyzer (Hitachi 912, Roche, Almere, The Netherlands) with kits from Randox (Crumlin, United Kingdom). Total protein was measured on an autoanalyzer (Hitachi 912, Roche, Almere, The Netherlands) with a BCA kit from Pierce (Etten Leur, The Netherlands). 

Total heme oxygenase (HO) activity determinations in preweighed frozen samples (100 mg) of cardiac lobes of the right lungs were done according to established procedures [18–20]. In summary, preweighed tissue samples were defrosted on wet ice, immediately thoroughly diced with surgical scissors, and sonicated in four volumes of phosphate-buffered saline, pH 7.4 (PBS) with one-second pulses from a Microson ultrasonic cell disrupter (Model XL 2000, Misonix, Farmingdale, NY, USA) with an 1/8-inch diameter microtip probe, operated at 50% power (10 Watt) for no more than 15 sec to obtain homogeneous suspensions. Sonicates (20 *μ*L) were incubated with equal volumes of NADPH (4.5 mM) and methemalbumin (50 *μ*M/11.2 mM) for 15 min at 37°C in 2 mL CO-purged, septum-sealed amber glass vials. Blank reaction vials contained PBS in place of NADPH. Reactions were terminated by the addition of 5 *μ*L 30% (w/v) sulfosalicylic acid and the vials were placed in ice. The amount of CO generated during the reaction and released into the vial head space was quantitated by gas chromatography with a Reduction Gas Analyzer (RGA2, Trace Analytical, Menlo Park, CA, USA). HO activity is expressed as picomoles CO generated hr/mg fresh weight (or per mg protein). Protein concentrations in 1 : 100 dilutions of tissue sonicates were determined using the Protein Assay kit (Bio-Rad, Hercules, CA, USA).

### 2.6. Cardiovascular Analysis

#### 2.6.1. Plasma Analysis

Cell differentials were determined in ethylenediaminetetraacetic acid (EDTA; Terumo Europe, Leuven, Belgium), and anticoagulated blood was analyzed in an H1- E Multi-Species Haematology Analyser (Bayer, Mijdrecht, The Netherlands). The following parameters were measured: white blood cell (WBC) and red blood cell (RBC) concentrations, hemoglobin (HGB) and platelet concentrations (PLT), the mean platelet volume (MPV), and the haematocrit value (HCT). The mean platelet component (MPC), mean cell hemoglobin (MCH), mean cell hemoglobin concentration (MCHC), red blood cell distribution width (RDW), and hemoglobin distribution width (HDW) are also provided.

#### 2.6.2. Quantitative Real-Time PCR (rtPCR)

RNA from heart and aorta was extracted from pulverized Trizol homogenates according to the manufacturer's instructions. The RNeasy mini kit together with DNAse treatment (RNAse-free DNAse set, Qiagen) was used to purify total RNA from salts and residual DNA. Quantity and purity of RNA was evaluated using spectrophotometer at 230, 260, 280, and 320 nm. cDNA was prepared using the iScript cDNA Synthesis kit (BioRad, CA, USA), starting from 1 *μ*g of RNA. PCR primers for rat HO-1, inducible NO synthase (iNOS), Apurinic/apyrimidinic endonuclease/redox effector factor (Ape/Ref-1), and GAPDH (as a housekeeping gene) were designed using Primer express software (Applied Biosystems). These genes were selected as being relevant indicators of an oxidative stress response. Primer sequences for HO-1 were 5′-GGG AAG GCC TGG CTT TTTT-3′ (forward) and 5′-CAC GAT AGA GCT GTT TGA ACT TGGT-3′ (reverse), for iNOS 5′-AGG AGA GAG ATC CGG TTC ACA GT-3′ (forward) and 5′-ACC TTC CGC ATT AGC ACA GAA-3′ (reverse), for Ape/Ref-1 5′-GAA TGT GGA TGG GCT TCGA-3′ (forward) and 5′-ACA AGA TGT CTG GTG CTT CTT CCT-3′ (reverse), and for GAPDH 5′-TGA TTC TAC CCA CGG CAA GTT-3′ (forward) and 5′-TGA TGG GTT TCC CAT TGA TGA-3′ (reverse). Real-time PCR was performed with a MyiQ Single Colour real-time PCR detection system (BioRad) using SYBR Green Supermix (Biorad), 5 *μ*L diluted cDNA, and 0.3 *μ*M primers in a total volume of 25 *μ*L. PCR was conducted as follows: denaturation at 95°C for 3 min, followed by 40 cycles of 95°C for 15 seconds and 60°C for 45 seconds. After PCR, a melt curve (60–95°C) was produced for product identification and purity. PCR efficiency of both primer sets, as assessed by the use of cDNA dilution curves, was 100%. Data were analyzed using the MyiQ Software system (BioRad) and were expressed as relative gene expression (fold increase) using the 2^−ΔΔCt^ method [21].

#### 2.6.3. Tissue Factor Activity and Thrombin Generation Analysis

Snap frozen left lung tissues were freeze-dried for 3 days, pulverized and subsequently the powder was divided for RNA isolation and protein analyses. Fractions for protein analyses were dissolved in 50 mM n-octyl ß-D-glucopyranoside (Sigma-Aldrich) in HN-Buffer (25 mM HEPES, 175 mM NaCl, pH 7.7), vortexed, and centrifuged twice (10 min, 13000 rpm). Total protein content of the tissue homogenates was spectrophotometrically determined using the Biorad DC Protein Assay system according to the manufacturer's instructions (Bio-Rad Laboratories B.V., Veenendaal, The Netherlands).

TF activities in lung tissue homogenate were determined using a home-made activity assay as previously described ^E^[22]. In brief, dissolved lung tissue homogenates with a concentration of 1 mg/mL total protein were diluted 20 times in HN-buffer. A reference curve was prepared from Innovin (Dade Behring Holding GmbH, Liederbach, Germany), starting with 5 pM and diluted serially 7 times, also in HN buffer. Samples were incubated for 45 minutes at 37°C in the presence of recombinant factor VII (FVII) (Novo Nordisk, Bagsværd, Denmark), 0.2 mM 20/80 PS/PC vesicles, 1 U/mL Bovine factor X (Sigma-Aldrich), and 100 mM Ca^2+^. The formation of factor Xa was then measured kinetically using the chromogenic substrate S 2765 (Chromogenix, final concentration of 0.7 mg/mL diluted in 50 mM Tris-HCl, 175 nM NaCl, 30 mM Na2EDTA, pH 7.4) by measuring the OD at 405 nm each 15 seconds, for 15 minutes at 37°C.

Thrombin generation was measured by means of the Calibrated Automated Thrombogram (CAT, Thrombinoscope BV, Maastricht, The Netherlands) [23], which employs a low affinity fluorogenic substrate for thrombin (Z-Gly-Gly-Arg-AMC) to continuously monitor thrombin activity in clotting plasma. According to the manufacturer's instructions, measurements were conducted on 80 *μ*L full plasma in a total volume of 120 *μ*L and in the presence of 400 *μ*M fluorogenic substrate and 16 mM added CaCl_2_. In order to correct for inner-filter effects and substrate consumption, each thrombin generation measurement was calibrated against the fluorescence curve obtained in the same plasma with a fixed amount of thrombin/*α*2-macroglobulin complex (Thrombin Calibrator, Thrombinoscope BV). Fluorescence was read in a Fluoroskan Ascent reader (Thermo Labsystems OY, Helsinki, Finland) equipped with a 390/460 filter set, and thrombin generation curves were calculated with the Thrombinoscope software (Thrombinoscope BV). Five parameters were derived from the thrombin generation curves: lag time (min), endogenous thrombin potential or area under the curve (ETP, nM.min), peak height (nM), time to peak (min), and time to tail (min). Thrombin generation in rat plasma was determined in the presence of 1 pM TF (PPP Reagent Low, Thrombinoscope BV) and 4 *μ*M phospholipids (final concentrations). For determination of the thrombogenicity of rat lung tissue, 15 *μ*L of tissue homogenate (5 mg/mL total protein) was added to 80 *μ*L of platelet poor pooled human plasma (University Hospital Maastricht), which consisted of plasma from 80 healthy volunteers. Triggering of thrombin generation depended on tissue factor molecules present in the lung homogenate.

### 2.7. Statistical Analysis

Calculations were carried out using SPSS version 12.0 and Splus 6.2 for Windows. All data of biological effect parameters of the pulmonary system were log-transformed and, subsequently, a two-way analysis of variance (ANOVA) was performed. Two-way ANOVA analyses were used to assess differences due to exposure, time, day variance, and their interaction. *P* values <.05 are considered statistically significant. Data for DEE-treated animals were primarily expressed in terms of relative response to control levels.

## 3. Results

### 3.1. Pulmonary Effects

#### 3.1.1. Bronchoalveolar Lavage Fluid-Analysis

To evaluate the sequence of events following DEE-induced oxidative stress, key components of the anti-oxidant defense system as well as markers for inflammation were analyzed in BALF (Table 3). No signs of acute cytotoxicity were observed as indicated by lack of increased LDH and ALP levels. The only significant cytotoxic effect, that is, ALP increase, was noted at 18 h, where at the later time points (24, 48, and 72 h) slightly decreased values were observed, indicating the absence (or recovery) of epithelial cell damage. LDH levels were not affected by DEE exposure, suggesting maintained membrane integrity. Albumin in fluid obtained from the BALF, as an indicator of permeability of the alveolar barrier, was not altered upon exposure to DEE and not different among all investigated time points. As markers for the anti-oxidant defense response, the levels of the anti-oxidants UA, total glutathione, the GSH/GSSG ratio, and heme oxygenase-1 (HO-1) were measured. Glutathione levels were affected by DEE exposure, showing a decrease in the GSH and GSH/GSSG ratio levels at the 18 h time point. In addition UA was increased in DEE-exposed animals at 24 h. The level of the anti-oxidant enzyme HO-1 was increased by the DEE exposure at 24, 48 and 72 h. In general no effects were observed in total cell numbers in the BALF (data not shown). Proinflammatory cytokines IL-6 and TNF-*α* where both significantly increased at 48 h post-exposure. However, only total cell concentrations in the BALF were slightly decreased after 24 and 48 hours post-DEE exposure compared to the control group, which was mainly caused by a decrease in macrophages. No increase in PMN or lymphocytes due to DEE exposure was observed at any time point. 

#### 3.1.2. Lung Homogenate Analysis

**Figure 1:**
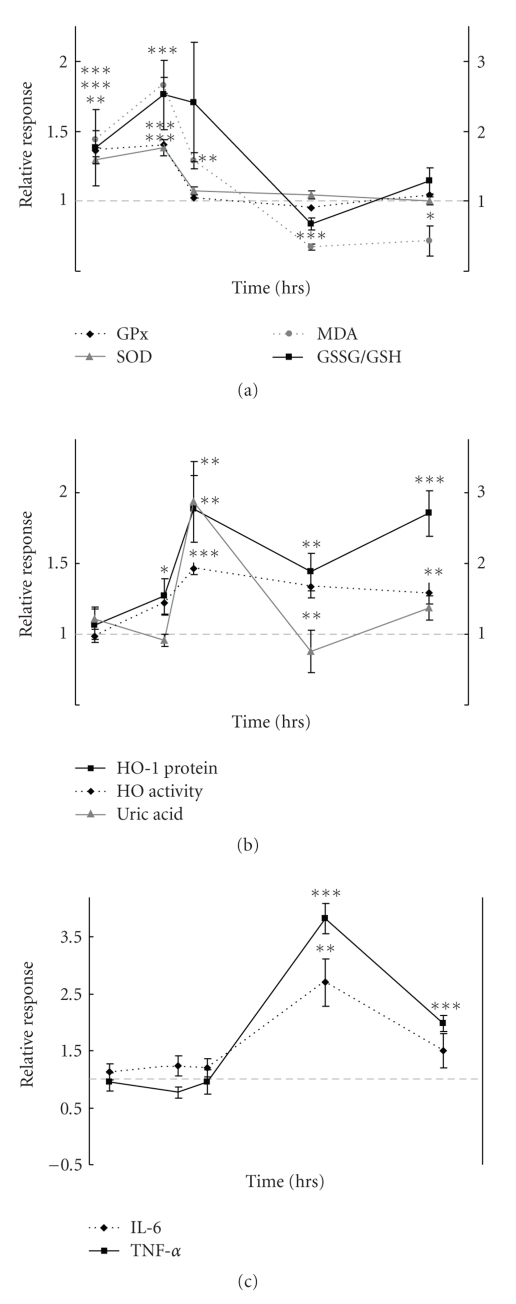
Oxidative stress and inflammation responses in samples from rats exposed to DEE. Relative response of markers, as depicted in legend boxes, at various time points (*t* = 4, 18, 24, 48, and 72 h) after termination of a 2-hour exposure of rats (*n* = 10 per time point and exposure) to DEE. (GSSG/GSH response is indicated by right y-axis). Relative response is defined as the mean value of the DEE exposed group divided by the mean value of the sham exposed group at the same time point. Error bars indicate the standard error of the mean, corrected for the error introduced by the normalization. *, **, *** significantly different from control at *P* < .05, <.01, <.001, respectively.

SOD, GPx and total HO activities were measured as important anti-oxidant enzymes responsible for the defense against damaging oxidation processes in the lungs such as lipid oxidation (Table 4). Increased GPx and SOD activities were observed between 4 and 18 h. Total HO activity was increased at 24 h in rats exposed to DEE and remained elevated during the next 48 h. Increased MDA levels were already noted at 4-hour after termination of the DEE exposure and returned to baseline values within 24 h.  Figure 1 illustrates the most indicative changes over time of anti-oxidant defense and inflammatory markers after DEE exposure.

### 3.2. Cardiovascular Effects

#### 3.2.1. Blood Analysis

Exposure-related effects were found for hematological parameters, that is, mean platelet volume (MPV) and mean platelet component (MPC), as a measure of platelet density. MPV was increased at 24 h and decreased at 18, 48, and 72 h. MPC was increased at 24 and 48 h and decreased at 4, 18, and 72 h. In addition, absolute numbers and percentage of circulating neutrophils were increased by 40% at 4 h. CC16 plasma levels, a marker of the integrity of the air-blood barrier, were not altered upon DEE exposure (data not shown).

#### 3.2.2. mRNA Quantification

In order to reveal cardiovascular oxidative effects of DEE inhalation, mRNA levels of oxidative stress responsive genes HO-1, Ape/Ref-1 and iNOS were evaluated in heart and aorta. In general, only effects at 4 h postexposure were observed and not at 24 h (Table 5). In particular, a mild increase was observed for genes HO-1 and Ape/Ref-1 in aortic tissue. No effects on gene expression were observed in heart tissue. 

#### 3.2.3. Tissue Factor (TF) Activity and Thrombin Generation Analysis

**Figure 2:**
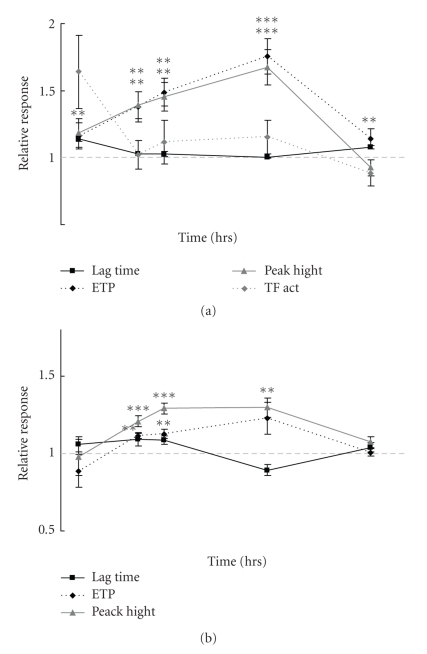
Relative response of TF activity (only in lung tissue), thrombogenicity lag time, ETP, and peak in lung homogenate tissue (a) and in plasma (b) at various time points (*t* = 4, 18, 24, 48 and 72 h) after termination of a 2 h exposure of rats (*n* = 10 per time point and exposure) to DEE. (Uric acid response is indicated by right *y*-axis). Relative response is defined as the mean value of the DEE exposed group divided by the mean value of the sham exposed group at the same time point. Error bars indicate the standard error of the mean, corrected for the error introduced by the normalization. *, **, *** significantly different from control at *P* < .05, <.01, <.001, respectively.

The lung-specific TF activity was significantly increased shortly after the termination of the DEE exposure (4 h) (Figure 2 upper panel). At later time points after DEE exposure, there was no significant change in TF activity observed.

The overall lung-specific thrombogenicity, as assessed by means of thrombin generation in normal pooled plasma, was increased due to DEE exposure. Both the ETP and peak height were increased at 4, 18, 24, and 48 h upon DEE exposure (Figure 2 lower panel). The lung-specific thrombogenicity reached its maximum at 48 h with up to 70% increased ETP and peak height. The increase in TF activity was not reflected by a decreased lag time, as expected (Figure 2). The increase in overall lung-specific thrombogenicity was reflected in plasma: both ETP and peak height followed the increase observed in lung tissue with a maximum at 48 h. Overall, plasma thrombin generation was increased by almost 30%, indicating a thrombotic state.

## 4. Discussion

The ability of PM to increase the intracellular production of ROS, thereby inducing oxidative stress, has been studied *in vitro* and *in vivo *[17, 24, 25]. However, the sequence of events commencing with oxidative stress and subsequently leading to adverse effects *in vivo* after a short-term inhalation exposure has hardly been studied at relevant concentrations. The present study was designed to determine the time course for cardiopulmonary health effects of DEE in rats, especially in relation to the oxidative stress response. Our data demonstrate that in the respiratory system the effect on the anti-oxidant defense system preceded a local inflammation and local and systemic procoagulant response; at 18 h the GSH/GSSG ratio was decreased, and the activity of the anti-oxidant enzymes GPx and SOD was increased, MDA levels were increased, at 24 h HO-1 protein and HO activity levels were increased, the amount of the anti-oxidant uric acid was increased, whereas an increase of the inflammatory markers IL-6 and TNF-*α* was only seen at the latest time points of investigation. This latter observation together with the absence of a PMN influx suggests that there is a delayed inflammatory response which may have its maximum after 72 h.

**Figure 3:**
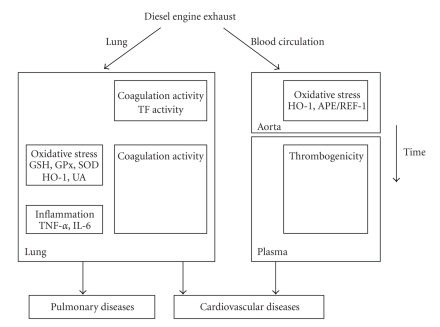
Hypothetical mechanism of the effect of diesel engine exhaust on the cardiopulmonary system.

Lung TF activity was increased at 4 h after exposure, whereas the overall thrombogenicity measured in lung tissue was increased from 18 h onwards. In the cardiovascular system, oxidative effects were only and very clearly observed at 4 h. Surprisingly, the plasma procoagulant state was increased at 18, 24, and 48 h after DEE exposure and followed the pattern observed for lung thrombogenicity. These results suggest that both the lung and the circulating blood have been exposed to chemicals from DEE resulting in time events which may lead to cardiopulmonary responses, as depicted in Figure 3.

Eighteen hours after the DEE exposure, a decrease in the GSH/GSSG ratio was observed which was caused by a decrease in GSH. This is in agreement with *in vitro* studies which have shown a decrease in the intracellular GSH/GSSG ratio in RAW264.7 cells after 16 h exposure of CAP, for fine and ultrafine fractions and an unchanged effect for the coarse fraction compared to the control [26]. In contrast, other studies have reported an increase in GSH after 1, 2, and 4 days of ozone-treated rats exposed to DEE, whereas monocrotaline treated rats did not show a change in GSH after exposure to DEE. In general it has been shown that minimal declines in the GSH/GSSG ratio, representing lower levels of oxidative stress, induce activation of anti-oxidant enzyme HO-1 and reflect the first tier of the oxidative stress response. Next steps in the hierarchical oxidative stress response are reflected by even greater declines in GSH/GSSG ratios and activation of NF-*κ*B and MAPK signaling pathways followed by an inflammation response. The final tier of oxidative stress response leads to cytotoxic effects [5].

We have studied the activity of three different anti-oxidant enzymes in lung homogenate after exposure of DEE. GPx and SOD are both early responders, and show increases in activity at the 4 h and 18 h time points. In contrast, high dose (1.5, 7.5 and 37.5 mg/kg) instillation studies showed decreased GPx and SOD values 24 h after one-time exposure [27]. These differences suggest the existence of a different tier in the oxidative stress response for both studies, most likely caused by differences in the time point of analyses and applied dose of oxidative stress. Our data indicate that HO-1 responded 18 h and 24 h after the DEE exposure. This is the first time that a quantitative assay has been used to measure the HO activity by CO production in lung tissue after DEE exposure. The assay provides a quantitative measure of the oxidative stress response at different time points after DEE exposure. Peak HO activity correlated well with the maximum for HO-1 protein content at the 24 h time point in the BALF. Measurements of mRNA HO-1 levels in heart and aorta showed an increase upon DEE exposure at 4 h but not at 24 h.

DEE exposure led to about a 50% increase in lipid peroxidation at the 18 h time point after exposure, as measured by the formation of MDA. This is in agreement with a study by Rhoden et al. showing that a 5 h 1060 *μ*g/m^3^ PM exposure led to about twofold increase in the levels of thiobarbituric acid reactive substances [17]. Our data suggest that a 2-hour DEE exposure of 1.9 mg/m^3^ results in a hierarchical oxidative stress response in which the first tier, indicated by an anti-oxidant defense, is reached between 4 and 24 h after exposure and the second tier after 48 and 72 h of exposure, indicated by an inflammatory response marked by increased levels of TNF-*α* and IL-6. This study therefore shows that different stages in oxidative stress are not only affected by dose increments as described by Li et al. [5], but also by time after exposure.

Despite clear increases of PMN in total plasma 4 h post exposure, no increased numbers of PMN inflammatory cells were observed in the airways. This is consistent with other studies in which the number of pulmonary PMN was not affected after a single 6-hour exposure to DEE (9 mg/m^3^) in compromised rats [28], and a 3 h exposition to 110–350 *μ*g/m^3^ concentrated PM [29].

The changes in values of the hematological indices MPV and MPC found after exposure to DEE are noteworthy and in line with a previous studies noting significant alterations in these indices [30]. These observations are therefore in agreement with the hypothesis that particles can affect hematological indices [31]. Increased MPV at 24 h may indicate an increase in platelet reactivity, via platelet stimulation or rate of platelet production. It has been observed that a reduction of MPC may be used to detect *in vitro* platelet activation [32]. However, since the observed differences are very small and opposite in sign at 24 and 48 h, it is questionable whether the changes are biologically relevant. In addition, thrombogenic effects after DEE exposure were observed. First of all, a procoagulant effect was observed in lung tissue as indicated by increased TF activity and thrombin generation upon DEE exposure. Increased thrombin generation is most likely the resultant of increased TF and decreased thrombomodulin expression, due to inflammatory stimulation [33]. Second, the overall plasma haemostatic balance was shifted towards a procoagulant state. A recent study suggested that this plasma effect may be IL-6 mediated [3]. IL-6 is known to mediate procoagulant effects in blood following endotoxin exposure to humans [34] and these effects are also TF dependent. Whether the systemic thrombogenicity inflicted by PM in our study was mediated by TF or the result of other pathways cannot be directly derived from our data. An argument against a cytokine (e.g., IL-6) mediated procoagulant activity in blood is the observation that the procoagulant response preceded the proinflammatory effects at 48 h. Such a time sequence may be more compatible with a direct effect of DEE on blood coagulation, through translocation of ultrafine particles. Indeed, incubation of PM alone in plasma showed a factor XII-mediated thrombin generation (Spronk et al., unpublished data). 

In a previous study in which we exposed mice to the oxidative stressor ozone we found that inflammation in the lung was followed by a procoagulant reaction [35]. The discrepancy between these studies might indicate that in the present study the procoagulant reaction is caused by direct effects of the ultrafine DEE particles, rather than indirect effects due to inflammation. A recent study by Ito et al. [36] showed similar effects in a 4-day 0.6–1.5 mg/m^3^ PM exposure study, wherein lung inflammation effects were low and not significant, but effects of the exposure on markers in the heart related to cardiovascular diseases were prominent. It is therefore suggested that soluble chemicals adsorbed on PM (or gaseous components in the mixture) might reach the heart directly and confer oxidative stress to the cardiovascular system. The increased expression levels of HO-1 and Ape/Ref-1 in aortic tissue at 4 h after DEE exposure in our study also indicate an acute and transient systemic response. Since these acute systemic effects are observed in the absence of a clear pulmonary response, this suggests a direct effect upon inhalation, caused by rapid translocation of DEE components (ultrafine particles, organics, volatile compounds) into the circulation. Ito et al. [36] found a similar significant up-regulation of HO-1 mRNA cardiac expression as a result of oxidative stress by PM on the heart, as supported by previous *in vivo* and *in vitro* studies [25, 26].

In conclusion, the present *in vivo* inhalation study with DEE showed signs of anti-oxidant defense at 18–24 h preceding a proinflammatory response at 48 h, supporting the hierarchical oxidative stress model. Procoagulant reactions and changes in expression of anti-oxidant defense genes were observed already after 4 h, which might suggest that DEE components are immediately translocated by plasma to aorta. However this effect can also be mediated by endogenous factors from the lung that were not measured in our study or were not associated with inflammation. Which pathway occurs *in vivo* remains to be investigated.

## Figures and Tables

**Table 1:** Chemical composition and concentrations of diluted DEE.

Parameter	Unit	DEE
Gravimetric mass	(mg/m^3^)	1.9
Particle count	(10^3^/cm^3^)	4125
CO	(ppm)	14.2
NO	(ppm)	1.4
NO_2_	(ppm)	0.9
NO_*x*_	(ppm)	2.4
OC	(*μ*g/m^3^)	423
EC	(*μ*g/m^3^)	48
TC	(*μ*g/m^3^)	471
NH_4_	(*μ*g/m^3^)	3.4
Cl	(*μ*g/m^3^)	0.9
NO_3_	(*μ*g/m^3^)	0.0
SO_4_	(*μ*g/m^3^)	6.6
VOC	(*μ*g/m^3^)	529

**Table 2:** Metal and PAH concentrations in diluted DEE.

Parameter	DEE (*μ*g/m^3^)
Cu	0.0189
Zn	0.6393
Rb	0.0529
Sr	0.0370
Ag	0.2002
Cd	0.2758
In	0.1848
La	0.2598
Hg	0.1314
Pb	0.3082
PAH	224

**Table 3:** Time course for health effect parameters measured in lung lavage fluid of F344 rats after DEE exposure or clean air as a control, represented as mean and 95% *c*.*i*. value (*n* = 10). *, **, *** significantly different from control at *P* < .05, <.01, <.001, respectively.

		*T* = 4 h		*T* = 18 h		*T* = 24 h		*T* = 48 h		*T* = 72 h	
Parameter		Control	DEE	Control	DEE	Control	DEE	Control	DEE	Control	DEE
ALP	Mean	54.7	50.4	47.9	72.6***	57.3	44.7*	69.5	47.1**	62.8	43.7**
	95% *c*.*i*.	48.9–60.5	43.8–57.0	37.0–58.8	63.8–81.4	47.0–67.7	37.3–52.1	57.9–81.1	35.2–59.0	53.7–72.0	35.3–52.0
LDH	Mean	67.1	73.5	67.6	71.1	72.3	68.4	89.3	67.9*	74.6	67.5
	95% *c*.*i*.	58.2–75.9	65.1–82.0	63.4–71.9	62.3–79.9	63.8–80.7	62.1–74.7	71.6–107.0	60.9–74.9	66.7–82.4	61.9–73.1
Albumin	Mean	204	187	209	198	191	179	201	180	195	184
	95% *c*.*i*.	185–222	165–209	188–230	182–214	169–213	157–201	173–229	164–196	184–206	171–196
NAG-B	Mean	1.75	2.52*	1.93	2.01	1.87	1.71	2.00	2.03	2.26	2.17
	95% *c*.*i*.	1.51–1.98	2.11–2.93	1.70–2.16	1.75–2.26	1.63–2.11	1.49–1.93	1.70–2.31	1.68–2.37	1.97–2.54	1.81–2.53
CC16	Mean	0.753	1.138	1.011	1.153	1.141	0.946	1.022	1.065	0.906	1.293
	95% *c*.*i*	0.655–0.850	0.931–1.344	0.852–1.170	0.977–1.329	1.013–1.269	0.811–1.081	0.858–1.185	0.835–1.296	0.693–1.119	1.089–1.497
UA-B	Mean	0.32	0.39	0.55	0.5	0.35	0.99**	0.92	0.7	0.46	0.63
	95% *c*.*i*.	0.26–0.38	0.28–0.49	0.12–0.98	0.40–0.61	0.21–0.49	0.54–1.44	0.39–1.46	0.07–1.33	0.34–0.58	0.46–0.81
GSH	Mean	0.78	0.66	0.91	0.52**	0.87	0.76	1.1	1.01	0.82	0.75
	95% *c*.*i*.	0.65–0.92	0.49–0.84	0.70–1.11	0.39–0.66	0.74–1.00	0.52–1.01	0.88–1.33	0.690–1.338	0.62–1.02	0.546–0.950
GSH/GSSG	Mean	6.86	5.44	5.96	2.77	5.44	4.03	5.21	6.70	4.39	3.48
	95% *c*.*i*.	1.84–11.88	2.76–8.12	3.25–8.68	1.23–4.32	4.28–6.60	1.16–6.89	3.47–6.94	5.54–7.87	3.11–5.66	2.18–4.77
HO-1	Mean	0.146	0.155	0.160	0.202	0.144	0.271**	0.156	0.224**	0.147	0.271***
	95% *c*.*i*.	0.135–0.157	0.121–0.190	0.135–0.184	0.163–0.242	0.124–0.164	0.204–0.338	0.132–0.180	0.184–0.263	0.123–0.170	0.226–0.317
IL-6	Mean	19.16	21.79	18.70	23.09	23.15	27.85	18.84	50.90**	25.25	37.85
	95% *c*.*i*.	11.0–27.3	16.6–27.0	5.7–31.7	16.8–29.4	10.5–35.8	20.5–35.2	6.5–31.2	35.3–66.5	16.5–34	22.8–52.9
TNF-*α*	Mean	76.23	72.47	74.77	57.47	49.70	47.09	57.82	221.53***	94.03	186.50***
	95% *c*.*i*.	49.2–103.2	48.6–96.3	56.0–93.5	43.4–71.5	32.2–67.2	26.6–67.6	33.1–82.6	191.4–251.7	74.6–113.5	160.1–212.9

**Table 4:** Time course for protein-corrected health effect parameters measured in lung homogenate of F344 rats after DEE exposure or clean air as a control, represented as mean and 95%* c.i.* value (*n* = 10, except for HO activity *t* = 4, 18 and 24 h were *n* = 5). *, **, *** significantly different from control at *P* < .05, <.01, <.001, respectively.

		*T* = 4 h		*T* = 18 h		*T* = 24 h		*T* = 48 h		*T* = 72 h	
Parameter		Control	DEE	Control	DEE	Control	DEE	Control	DEE	Control	DEE
GPx activity	Mean	16.8	22.8***	17.1	24***	19	19.4	21.1	20.1	18.8	19.6
	95% *c.i. *	16.0–17.6	21.8–23.8	16.6–17.7	23.1–24.9	17.5–20.4	18.8–20.0	19.8–22.3	19.3–20.8	17.9–19.7	18.4–20.8
SOD activity	Mean	1.18	1.53***	1.19	1.65***	1.23	1.32	1.35	1.41	1.29	1.29
	95% *c.i. *	1.09–1.26	1.46–1.60	1.14–1.25	1.50–1.80	1.09–1.37	1.24–1.40	1.25–1.46	1.32–1.50	1.20–1.37	1.20–1.37
MDA	Mean	0.198	0.284**	0.165	0.301***	0.186	0.24**	0.202	0.137***	0.231	0.166*
	95% *c.i. *	0.153–0.243	0.256–0.312	0.145–0.185	0.280–0.322	0.162–0.210	0.217–0.263	0.184–0.220	0.126–0.148	0.215–0.247	0.108–0.224
HO activity	Mean	2.47	2.43	1.75	2.13*	1.78	2.60***	1.48	1.98	1.33	1.71**
	95% *c.i. *	2.15–2.78	*2.32–2.55*	1.59–1.91	1.85–2.40	1.56–2.00	2.45–2.76	1.28–1.68	1.74–2.21	1.19–1.46	1.51–1.91

**Table 5:** Gene expression ratios (*m* *e* *a* *n* ± *S* *D*) in rat aorta and heart tissue due to exposure to DEE as determined by quantitative real-time polymerase chain reactions.

	4 h aorta	4 h heart	24 h aorta	24 h heart
Ape/Ref-1	1.66 ± 1.00	0.96 ± 0.40	0.90 ± 0.25	0.92 ± 0.28
iNOS	0.91 ± 0.42	0.90 ± 0.21	1.00 ± 0.49	0.76 ± 0.25
HO-1	1.93 ± 0.96	0.85 ± 0.28	0.88 ± 0.43	0.89 ± 0.33
